# Allogenic blood patch pleurodesis for management of pneumothorax in a Cavalier King Charles Spaniel puppy with multiple pulmonary blebs and bullae

**DOI:** 10.1111/jvim.16465

**Published:** 2022-06-24

**Authors:** Conor Moloney, Antonella Puggioni, Myles McKenna

**Affiliations:** ^1^ School of Veterinary Medicine University College Dublin Dublin Ireland; ^2^ Present address: Zoetis Inc. Cherrywood Business Park Loughlinstown Dublin Ireland

**Keywords:** pleural space disease, pneumothorax, respiratory, lung

## Abstract

A 9‐week‐old male intact Cavalier King Charles Spaniel was presented for evaluation of acute onset dyspnea caused by left‐sided pneumothorax. Thoracic computed tomography (CT) identified multiple pulmonary bullae and blebs in multiple lung lobes. Rupture of ≥1 pulmonary blebs or bullae, precipitated by low impact trauma, was the suspected cause of pneumothorax. A volume of 7.5 mL/kg of fresh whole blood was collected from a type‐matched donor dog and administered into the left pleural space using a thoracostomy tube. The pneumothorax was successfully resolved and no adverse effects of blood patch pleurodesis were noted. The dog was clinically normal 12 months later.

**Abbreviations**
CT: computed tomography
ELISA: enzyme‐linked immunosorbent assay
RI: reference interval
SpO2: peripheral capillary oxygen saturation

## 1 CASE DESCRIPTION

A 9‐week‐old male intact Cavalier King Charles Spaniel was presented to University College Dublin Veterinary Hospital for evaluation and management of acute onset of dyspnea. The dog had been in the owner's possession for 2 weeks and had no previous relevant medical history except for a fall from a chair 2 days before presentation. On physical examination, the dog was tachypneic, with a respiratory rate of 56 breaths per minute, and had a restrictive breathing pattern. Auscultation of the left hemithorax identified decreased lung sounds dorsally and increased bronchovesicular lung sounds ventrally. Auscultation of the right hemithorax and heart were normal. Body condition score was 4/9. The remainder of the physical examination was unremarkable.

Thoracic‐focused assessment by ultrasonography identified absence of a glide sign in the left hemithorax, consistent with free gas in the pleural space. A volume of 120 mL of air was aspirated from the left pleural space by ultrasound‐guided thoracocentesis, confirming the presence of a left‐sided pneumothorax. Venous blood gas analysis identified a mild respiratory acidosis attributed to hypoventilation (pH, 7.32; reference interval [RI] 7.35‐7.46; pCO_2,_ 53 mm Hg; [RI] 30‐43). Peripheral capillary oxygen saturation (SpO2) was 93%. Complete blood count and serum biochemistry indicated no clinically relevant abnormalities. Serum (Angio Detect [ELISA], Idexx Laboratories) and fecal tests (modified Baermann) were negative for *Angiostrongylus vasorum*.

**FIGURE 1:**
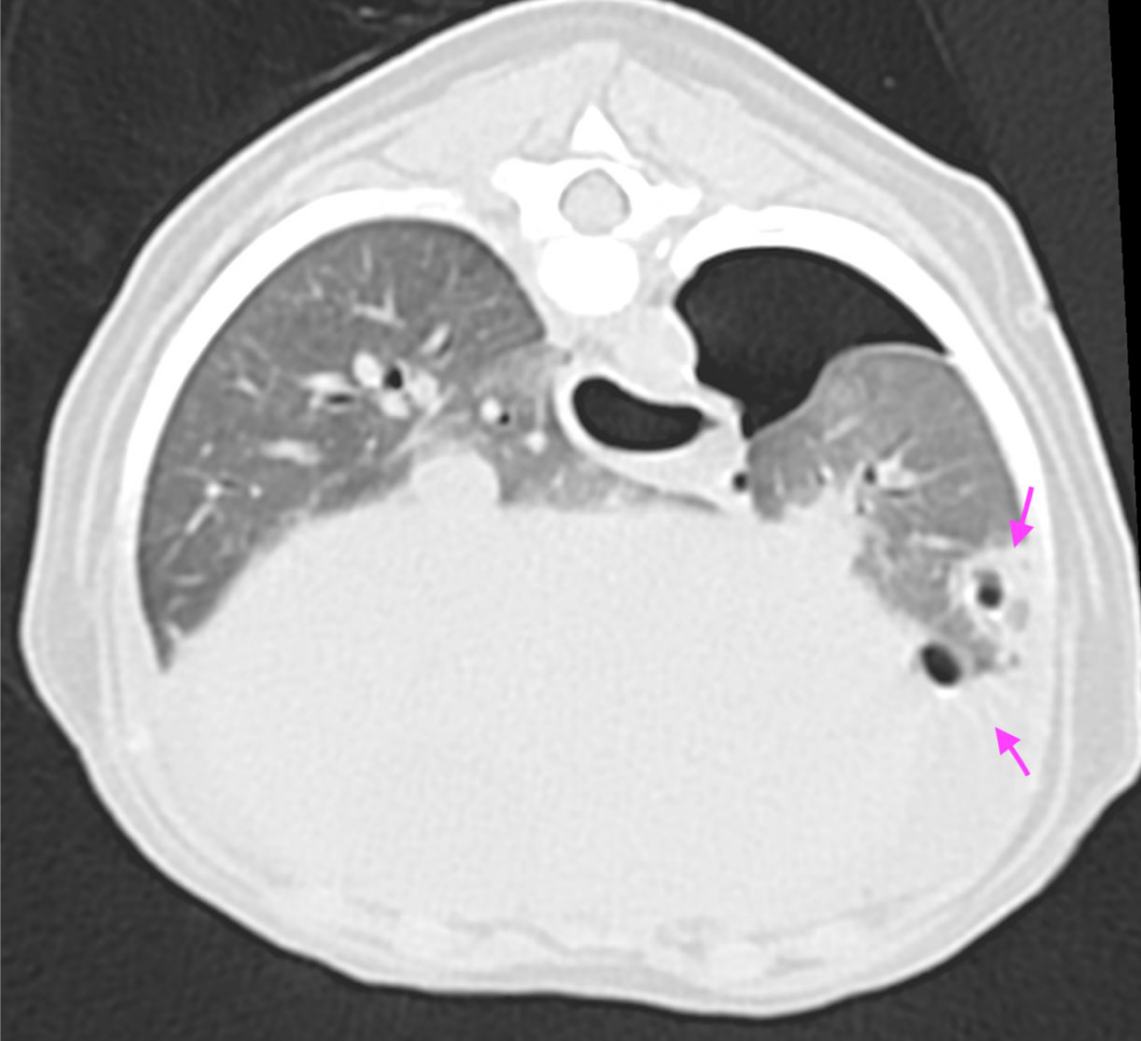
Transverse computed tomographic image of the thorax at the level of the left caudal lung lobe. Moderate amount of free pleural air is seen in the dorsal aspect of the left hemithorax. Two bullae are seen in the ventral aspect of the left caudal lung lobe. Both are surrounded by focal alveolar infiltration (pink arrows)

To further investigate the left‐sided pneumothorax, computed tomography (CT) of the thorax was performed under sedation (Somatom Scope, Syngo CTVC40, 16 slices Siemens, Germany). A moderate amount of pleural free air was seen in the dorsal left hemithorax, and a smaller amount was present in the right cranial hemithorax extending medially and mimicking pneumomediastinum. Several well‐defined structures of irregular shape and central gas attenuation consistent with pulmonary bullae were scattered throughout the mid‐caudal lung fields bilaterally. Dimensions of the bullae ranged from 2 to 6.5 mm; the largest bullae were located ventral to the caudal vena cava. Many of the smallest gas‐attenuating foci were located peripherally in a subpleural position, suggestive of pulmonary blebs. All of the bullae had well‐defined surrounding rims of soft tissue attenuation. At least 3 of the bullae in the ventral left caudal lung lobe were surrounded by areas of pulmonary parenchyma with increased attenuation consistent with focal alveolar infiltrate, suggestive of either consolidation or contusion. Similar lesions also were seen in other sections of both the left and right lung lobes (Figure 1). No evidence of pulmonary emphysema was identified.

**FIGURE 2:**
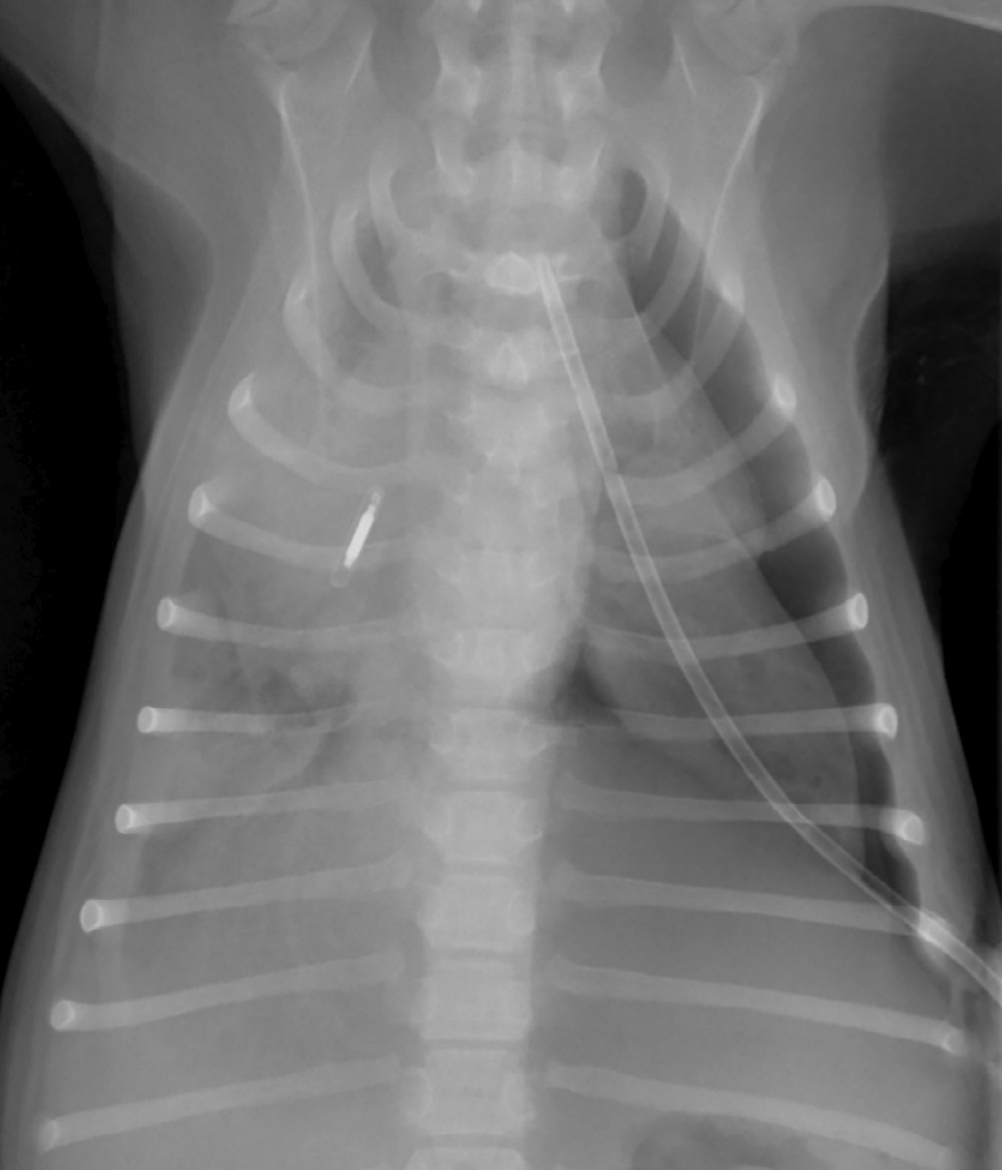
Dorsoventral radiograph of the thorax, taken after insertion of the thoracostomy tube. Marked pneumothorax is present on the left hemithorax with reduced volume of the left cranial and caudal lung lobes

**FIGURE 3:**
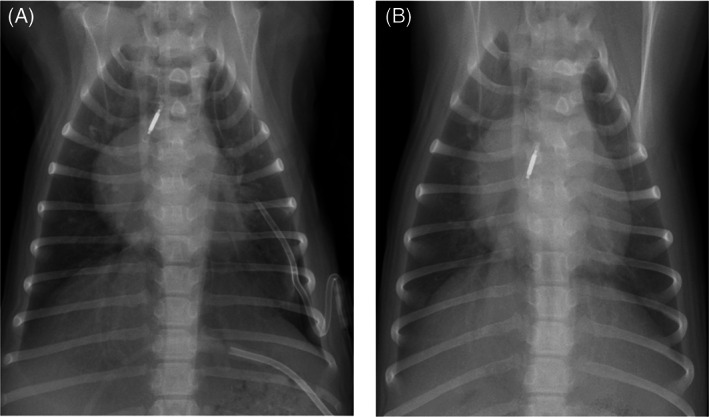
(A, B) Dorsoventral radiographs of the thorax taken at time of discharge and a week later. In (A), a small residual amount of free pleural air is still visible in the left costo‐diaphragmatic angle. In (B), there is no residual pneumothorax visible

To facilitate further management of the pneumothorax, a thoracostomy tube (14Ga Chest Tube, MILA International Inc, Florence, Kentucky) was placed in the 8th intercostal space in the left dorsal hemithorax. A single dorsoventral thoracic radiograph confirmed appropriate thoracostomy tube placement (Figure 2). Repeat pleural evacuation was required 5 times within 1 hour after thoracostomy tube placement to resolve clinical signs of recurrent tachypnea and increased respiratory effort. An average volume of 100 mL air was removed during each evacuation. Given the rapid recurrence of pneumothorax, we elected to perform allogenic blood patch pleurodesis. The dog was blood typed (Rapidvet‐H, DMS Laboratories Inc) and identified as being DEA 1 positive. Twenty mL of blood were drawn from the jugular vein of a blood type‐matched donor dog into a standard 30 mL syringe free of any additives or anti‐coagulant. This donated volume of blood was equivalent to 7.5 mL/kg for the recipient dog. By aseptic technique, the blood was immediately delivered through the in situ thoracostomy tube and into the pleural space of the patient, which was subsequently irrigated with 3 mL of nonheparinized sterile saline. The dog was gently rocked back and forth to facilitate distribution of the injected blood throughout the pleural space and to maximize contact with any sites of air leakage. The procedure was well tolerated, and no immediate complications were encountered. Respiratory rate and effort were closely monitored in the hours after the procedure to screen for potential recurrence of pneumothorax. Additionally, heart rate and rectal temperature were closely monitored to assess for signs suggestive of an acute transfusion reaction. Aspiration of the thoracostomy tube only was performed if the dog displayed signs of increased respiratory effort so as not to precipitate rupture of any of the other pulmonary bullae or blebs. Aspiration of the thoracostomy tube was required on 1 occasion 6 hours after blood patch pleurodesis because of a progressive increase in respiratory rate and effort, resulting in the evacuation of 60 mL air. Thereafter, the dog remained eupneic and no further pleural evacuation was required. The thoracostomy tube was removed 3 days after its placement. Repeat thoracic radiographs taken 24 hours after thoracostomy tube removal identified only a minimal residual left‐sided pneumothorax (Figure 3A) and the dog was discharged with no medications. On re‐evaluation 1 week later, the dog's owners reported it to be clinically normal since the time of discharge. Physical examination at this time identified no abnormalities and thoracic radiographs indicated no evidence of pneumothorax (Figure 3B). The dog remained clinically well 12 months after the time of presentation, based on a follow‐up telephone call.

## 2 DISCUSSION

We describe the occurrence of pneumothorax in a young dog with multiple pulmonary bullae and blebs. Pulmonary blebs are defined as accumulations of air within the layers of the visceral pleura, whereas pulmonary bullae are air‐filled spaces within the lung parenchyma that can result from destruction, dilatation and confluence of adjacent alveoli.^1^, ^2^ Spontaneous pneumothorax is defined as the development of pneumothorax in the absence of clinically relevant trauma.^3^ Although the dog was reported to have fallen off of a chair 2 days before presentation, spontaneous pneumothorax initially was assessed to be much more likely than traumatic pneumothorax because of the low impact nature of the trauma and detection of multiple pulmonary bullae and blebs on thoracic CT. However, the focal areas of alveolar infiltrate detected on CT could represent pulmonary contusions and as such traumatic pneumothorax caused by more severe unobserved trauma cannot be ruled out. Although the precise location of the site of air leakage could not be identified on the CT scan, the most likely explanation for this dog's pneumothorax is that the episode of low impact trauma precipitated rupture of ≥1 blebs or bullae, with subsequent leakage of air into the left pleural space.

Bulla or bleb rupture, and subsequent leakage of air from the alveoli into the pleural space, is the most commonly identified cause of spontaneous pneumothorax in dogs^1^, ^4^, ^5^ and is most commonly reported in large breed^1^, ^4^, ^6^ and in middle‐aged dogs.^1^, ^7^ Reports of pulmonary bullae and blebs in juvenile dogs are uncommon and most previously described cases have occurred in association with congenital lobar emphysema.^8^, ^9^, ^10^, ^11^, ^12^, ^13^, ^14^, ^15^ In congenital lobar emphysema, ≥1 lung lobes become hyperinflated, usually, but not always, as a result of congenital bronchial cartilage abnormalities. The associated bullae and blebs generally are present only in the emphysematous lung lobe or lobes.^8^, ^9^, ^10^, ^11^, ^12^, ^13^ Imaging findings consistent with congenital lobar emphysema (eg, hypoattenuation of affected lung parenchyma, mediastinal shift, flattened diaphragm^16^) were not present in the dog in our report, despite the presence of bullae and blebs in multiple lung lobes. Pulmonary bullae and pneumothorax in juvenile dogs also have been described in association with *Angiostrongylus vasorum* infection.^17^, ^18^ Although serological testing and fecal testing for *Angiostrongylus vasorum* were negative in this dog, the possibility of occult angiostrongylosis cannot be excluded.^19^ Bronchointerstitial pneumonia and neoplasia have been associated with pulmonary bullae or similar cystic pulmonary lesions in dogs.^20^, ^21^ Bacterial pneumonia was considered unlikely in this case given the acute presentation and rapid clinical improvement in the absence of antibiotic treatment, and neoplasia was considered very unlikely given the dog's young age. Other reported infectious causes of pulmonary bullae or similar lesions include *Blastomyces dermatidis*,^22^ *Paragnonimus* spp.,^23^ and *Dirofilaria immitis*.^24^ This dog had never left Ireland, a nonendemic area for all of these pathogens.

In human medicine, spontaneous pneumothorax associated with rupture of pulmonary blebs and bullae is reported rarely in children, with an increased incidence in males compared to females (4 : 1) and in individuals with low body mass index and in those with aesthenic features.^25^ Rapid growth of the vertical and horizontal dimensions of the thorax in adolescence are hypothesized to lead to overdistension of alveoli in peripheral areas of the lungs with subsequent bulla and bleb formation.^26^ Additionally, several rare diseases have been associated with the formation of pulmonary blebs or bullae in children, including Langerhans cell histiocytosis,^27^ Birt‐Hogg‐Dubé syndrome and other folliculin gene mutations,^28^, ^29^ and alpha‐1 antitrypsin deficiency.^30^ The dog in this case report had no specific clinical signs, clinicopathologic findings, or imaging findings that were suggestive of a similar underlying disease, but genetic testing and lung histopathology would be required to fully exclude this possibility. In the absence of evidence of underlying lung disease, the etiology of pulmonary blebs and bullae in this case was regarded as idiopathic.

Treatment options for management of pneumothorax associated with ruptured bullae or blebs include conservative management with placement of a thoracostomy tube and repeated thoracocentesis or continuous suction, blood patch pleurodesis, or surgical thoracotomy for identification and removal of the lung lobe from which air leakage has occurred.^1^, ^18^, ^31^, ^32^ Conservative management with repeated or continuous suction was not elected in this case because of the rapid recurrence of pneumothorax after drainage of the thoracotomy tube, the low reported success rate of conservative management^1^ and because of concerns that repeated thoracocentesis or continuous suction could cause some of the other bullae or blebs to rupture. Blood patch pleurodesis was elected as an initial treatment option over thoracotomy in this case because of the fact that multiple blebs and bullae were present, which may have complicated identification of the ruptured bleb or bulla intraoperatively. Additionally, blood patch pleurodesis represented a more conservative treatment option than thoracotomy, and therefore was preferable for a young puppy. Thoracotomy would have been considered if blood patch pleurodesis had been unsuccessful.

Blood patch pleurodesis for management of pneumothorax is believed to work by the mechanical action of coagulated blood adhering to the site of air leakage. In addition, the inflammation and fibrinous pleuritis that results from the presence of coagulated blood in the pleural space promotes the formation of adhesions and, eventually, pleurodesis.^33^, ^34^ Blood patch pleurodesis for management of pneumothorax has been described previously in humans, dogs, cats and, in research settings, in rats and rabbits.^18^, ^31^, ^32^, ^33^, ^34^, ^35^, ^36^, ^37^, ^38^ Existing reports of blood patch pleurodesis in dogs have involved autologous blood patch pleurodesis^18^, ^31^, ^32^ whereby autologous whole blood is injected into the pleural space. Collecting and administering 5 to 10 mL/kg (10% of body weight) of whole blood has been recommended for autologous blood patch pleurodesis in dogs.^18^, ^32^, ^33^ Autologous whole blood pleurodesis was not performed in this case because the dog was sedated, and because collection of 10% of blood volume was not considered safe in this dog because of its young age, small size and respiratory compromise. Allogenic blood patch pleurodesis, whereby the blood used is collected from a donor animal of the same species, previously has been reported in cats^37^ but to our knowledge has not been described in dogs. Allogenic blood patch pleurodesis was successful in preventing recurrence of pneumothorax in the dog in this report; it is uncertain whether conservative management without blood patch pleurodesis would have been successful. No adverse effects of the procedure were noted. Reported adverse effects of autologous blood patch pleurodesis in humans and dogs include transient fever, and rarely infection, empyema and pleural effusion.^31^, ^34^, ^35^, ^36^, ^39^, ^40^ No adverse effects were reported in a recent case report of 3 cats receiving allogenic blood patch pleurodesis.^37^ Although transfusion reactions have not been reported in animals receiving allogenic blood pleurodesis, given the paucity of reports of this procedure in the veterinary literature, the risk of an immunologic transfusion reaction must be considered. Blood typing both the donor dog and the recipient dog before the procedure is recommended. If repeat allogenic blood patch pleurodesis is performed, cross‐matching the donor and recipient dogs also should be considered. Documentation of additional cases in which allogenic blood pleurodesis is used will provide further data on the safety and efficacy of this procedure in dogs.

## CONFLICT OF INTEREST DECLARATION

Authors declare no conflict of interest.

## OFF‐LABEL ANTIMICROBIAL DECLARATION

Authors declare no off‐label use of antimicrobials.

## INSTITUTIONAL ANIMAL CARE AND USE COMMITTEE (IACUC) OR OTHER APPROVAL DECLARATION

Authors declare no IACUC or other approval was needed.

## HUMAN ETHICS APPROVAL DECLARATION

Authors declare human ethics approval was not needed for this study.
